# Bond Strength of Injectable Composites Under Different Enamel Treatments and Bonding Strategies

**DOI:** 10.1002/cre2.70464

**Published:** 2026-09-25

**Authors:** João Daniel Paganella Chaves, Elisa Souza Camargo, Rodrigo Nunes Rached, Evelise Machado de Souza

**Affiliations:** ^1^ Graduate Program in Dentistry, School of Medicine and Life Sciences Pontifícia Universidade Católica do Paraná Curitiba Brazil

**Keywords:** bond strength, composite resins, dental enamel, highly filled flowable composites, universal adhesive

## Abstract

**Objective:**

The effectiveness of enamel acid etching may be enhanced when combined with additional surface treatments, such as diamond bur grinding. However, there are no reports of the effect of enamel grinding and bonding strategies when a universal adhesive and highly filled flowable composites are used. The aim was to evaluate the effect of enamel grinding and adhesive strategy on the microshear bond strength (micro‐SBS) of injectable composite resins.

**Materials and Methods:**

One hundred sixty enamel specimens were randomly allocated into 16 groups according to enamel preparation (diamond bur grinding or no grinding), adhesive strategy (self‐etch or etch‐and‐rinse), and composite type (three injectable composites and one conventional composite). After 48 h of water storage, the specimens were tested for micro‐SBS in a universal testing machine (0.5 mm/min). Data were analyzed using three‐way ANOVA and Tukey's test for micro‐SBS data, and Fisher's exact test and Chi‐square test for failure mode (*α* = 0.05).

**Results:**

A significant main effect was observed for bonding strategy (*p* < 0.001), but not for enamel preparation (*p* = 0.042) and composite resin (*p* = 0.044). The etch‐and‐rinse strategy resulted in significantly higher bond strength than the self‐etch approach, irrespective of the enamel preparation, for all the resin composites (*p* < 0.05). Adhesive strategy was the most frequent mode of failure (35%), with self‐etching yielding a 70% rate of adhesive failures compared to 0% for the etch‐and‐rinse approach.

**Conclusions:**

The etch‐and‐rinse strategy improved enamel bond strength and eliminated adhesive failures for all composites tested, regardless of enamel grinding.

**Clinical Significance:**

Enamel should be etched before applying a universal adhesive for injectable composite restorations, as this increased bond strength and eliminated adhesive failures regardless of enamel grinding or composite type. Diamond bur grinding only reduced adhesive failures when the self‐etch mode was used.

## 1 Introduction

The development of flowable composite resins in the 1990s represented a significant advancement in dental restorative materials (Labella et al. 1999; Bayne et al. 1998). Initially, these composites exhibited a lower filler content (37%–53% by volume) compared with conventional paste‐like composite resins, resulting in higher fluidity and a reduced elastic modulus (Bayne et al. 1998; Baroudi and Rodrigues 2015). However, due to their limited mechanical properties and increased polymerization shrinkage, early flowable composites were primarily indicated for areas subjected to low occlusal stress (Baroudi and Rodrigues 2015).

With the evolution of flowable composites, new formulations with higher filler content, known as highly filled flowable or injectable composites, have emerged. These materials demonstrate improved fracture toughness and wear resistance (Sumino et al. 2013). A 36‐month clinical evaluation reported that highly filled composites perform similarly to conventional composites in direct posterior restorations (Kitasako et al. 2016). Nevertheless, a recent systematic review identified drawbacks in laboratory studies that may limit their clinical application in extensive cavities, load‐bearing areas, and cases involving severe tooth wear (Tzimas et al. 2025).

New application techniques for these composite resins have also been introduced, such as the mock‐up or silicone guide technique. This method employs a transparent silicone guide fabricated from a diagnostic wax‐up to build direct restorations without requiring tooth preparation (Coachman et al. 2020; Liaropoulou et al. 2025). This approach has expanded clinical indications to include restoration of fractured teeth, direct veneers, and reestablishment of occlusal vertical dimension (Liaropoulou et al. 2025). However, as this technique is relatively recent, long‐term clinical studies are needed to assess the efficacy of highly filled flowable composites and the injectable technique.

One of the challenges associated with the silicone guide technique is the absence of prior enamel preparation before acid etching, which may compromise the bond strength of restorative materials (Bourgi et al. 2024; Vinagre et al. 2015). To optimize adhesion, various enamel surface treatments and adhesive protocols have been investigated (Abdalla et al. 2010; Hoshika et al. 2018; Suzuki et al. 2021; Takeda et al. 2019). Phosphoric acid etching is widely used because it enhances wettability and increases surface roughness and surface free energy, thereby promoting micromechanical interlocking and improving bond strength (Amran et al. 2025; Zafar and Ahmed 2015).

Contemporary adhesives were developed to reduce technique sensitivity and simplify clinical procedures, leading to more practical alternatives (Miyazaki et al. 2014). In line with this evolution, universal adhesives have emerged as versatile options (Foly et al. 2025), allowing different etching strategies, including total‐etch, self‐etch, and selective‐etch approaches (Chen et al. 2015). A recent clinical trial demonstrated that universal adhesives perform comparably or better than conventional total‐etch adhesive systems (Lawson et al. 2015). However, there is evidence that enamel bond strength achieved with universal adhesives is compromised in the absence of phosphoric acid etching, possibly due to their limited etching capability (Cuevas‐Suárez et al. 2019).

The effectiveness of acid etching may be enhanced when combined with additional surface treatments, such as aluminum oxide abrasion (Rifane et al. 2024) or diamond bur grinding (Daher et al. 2021). Some studies report that bond strength to enamel prepared with diamond burs is comparable to other methods when conventional adhesive systems are used (Van Meerbeek et al. 2003). Conversely, other authors suggest that diamond bur roughening may serve as a complementary technique, particularly when self‐etch adhesive systems are applied (Abdalla et al. 2010; Hoshika et al. 2018; Takeda et al. 2019). This procedure removes the outer aprismatic enamel layer, which is highly mineralized and acid‐resistant, thereby exposing the underlying prismatic enamel (Bourgi et al. 2024). Additionally, surface roughening increases the available bonding area and enhances the infiltration and demineralization potential of self‐etch primers, resulting in improved bond strength (Hoshika et al. 2018; Takeda et al. 2019). However, there is no report of an investigation of the effect of enamel grinding and etch‐and‐rinse versus self‐etch approaches when highly‐filled composites are used.

Therefore, the aim of this study is to evaluate the effect of enamel acid etching and grinding with a diamond bur on the microshear bond strength (µSBS) of three injectable and one conventional nanofilled composite resins. The null hypotheses were that: (1) enamel surface treatment would not affect µSBS; (2) bonding strategy would not affect µSBS; (3) composite type would not affect µSBS.

## 2 Materials and Methods

The sample size calculation was performed using an independent‐samples *t*‐test, considering *α* = 0.05 and 80% statistical power. The analysis indicated that 10 specimens per group would be sufficient to detect a minimum difference of approximately 2.5 MPa, assuming a standard deviation of 2.0 MPa. The calculation was performed using G*Power software, version 3.1.

Eighty sound maxillary and mandibular molars, extracted less than 6 months prior to testing, were collected from the Institutional Biobank and stored in 0.5% chloramine T solution at 4°C. The roots were sectioned 1 mm below the cement‐enamel junction using a water‐cooled diamond disc in a precision cutting machine (Isomet 1000, Buehler, Lake Bluff, IL, USA). Each crown was then sectioned longitudinally through the central groove, creating mesial and distal halves from the same tooth and resulting in a total of 160 specimens. The mesial and distal halves from each tooth were randomly assigned to different experimental conditions according to a factorial design comprising four composite types, two enamel surface conditions, and two adhesive strategies (4 × 2 × 2 = 16 experimental groups; *n* = 10 specimens per group). Random allocation was performed using computer‐generated random numbers in Microsoft Excel, ensuring that the two specimens obtained from the same tooth were assigned to different treatment combinations. All procedures were performed by only one experienced operator.

Each enamel half was horizontally positioned into a 25‐mm‐height and 20‐mm‐diameter polyvinyl chloride (PVC) ring and embedded in acrylic resin, leaving a 1‐mm‐height top enamel surface exposed. Next, the enamel surface was wet‐polished with #600, #1000, and #1200 grit silicon carbide (SiC) abrasive papers and then cleaned for 20 min in an ultrasonic bath with distilled water to remove debris.

Enamel preparation:

- No grinding: The enamel surface was left untreated.
- Diamond bur grinding: Gentle grinding with a water‐cooled tapered diamond bur (#2135, KG Sorensen, Barueri, SP, Brazil), applied lightly in back‐and‐forth movements for 5 s without exposing dentin. The diamond bur was replaced after every 5 specimens. All enamel surfaces were air‐dried and covered with double‐sided adhesive tape with a 1‐mm‐diameter hole to delimit the bonding area.

Bonding strategies:

- Self‐etch: No prior phosphoric acid application.
- Etch‐and‐rinse: Enamel was etched with 35% phosphoric acid gel (Ultra‐Etch, Ultradent Prod. Inc., South Jordan, UT, USA) for 30 s, followed by rinsing with an air‐water spray for 10 s and gentle air‐drying. For both adhesive application modes, the universal adhesive (Scotchbond Universal, Solventum, St. Paul, MN, USA) was actively applied for 20 s, followed by a gentle air‐stream for 5 s, and then light‐cured for 20 s using a high‐irradiance LED curing unit (Valo Grand Cordless, Ultradent, South Jordan, UT, USA) with an approximate output of 1000 mW/cm^2^.

**Figure 1:**
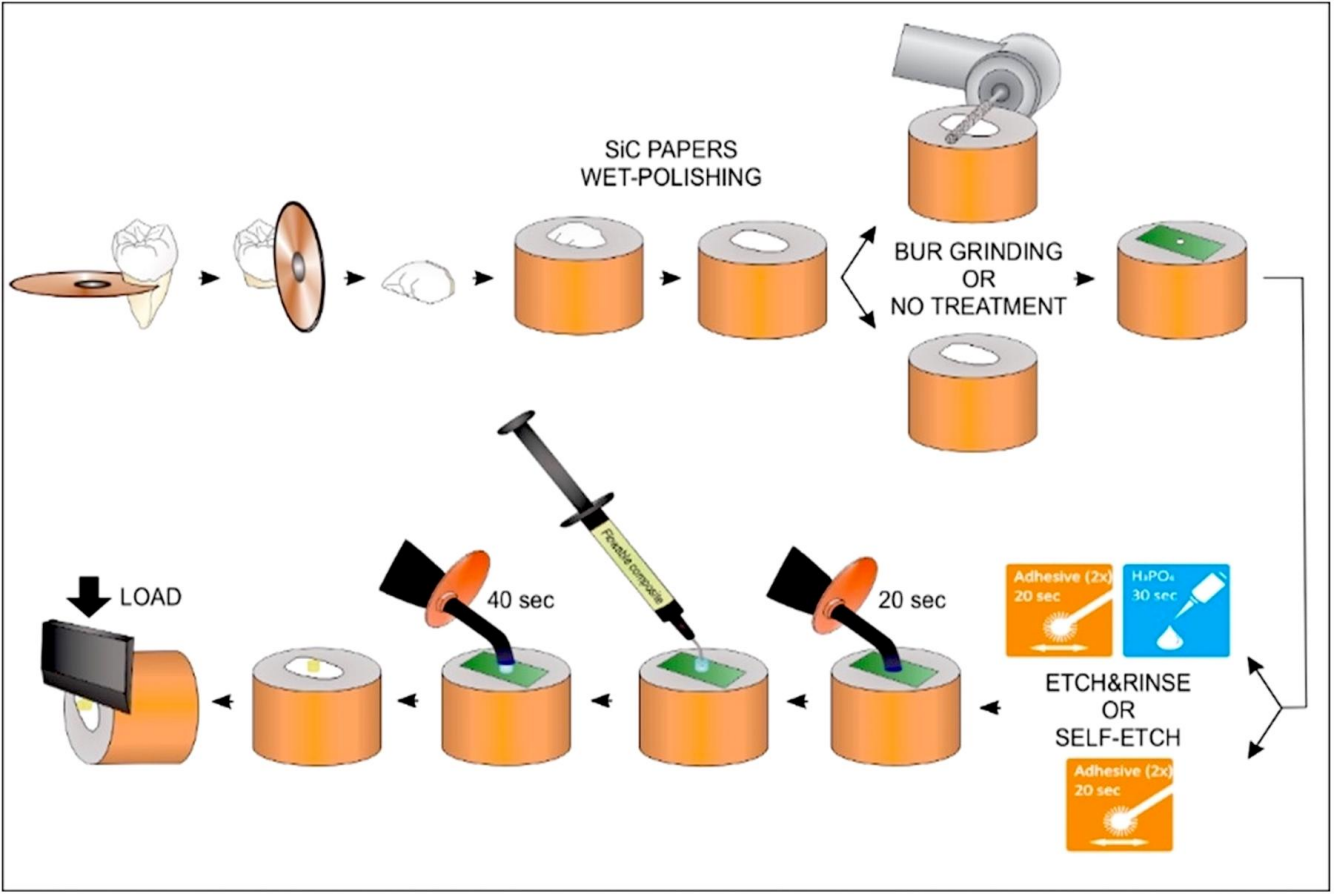
Schematic illustration of the experimental design. Enamel specimens were prepared using SiC wet‐polishing, followed by bur grinding or no surface treatment. Adhesive protocols included etch‐and‐rinse or self‐etch strategies prior to composite build‐up and shear bond strength testing.

Resin composite insertion: A translucent polyethylene tube (3.5 mm height × 1.5 mm external diameter × 1.0 mm internal diameter) was positioned over the bonding area of each specimen and filled with one of the four resin composites evaluated (three highly filled flowable composites and one conventional composite; Table 1). The injectable composites were delivered directly into the tube through their metallic needle tips, whereas the conventional composite was inserted and condensed with a fine hand instrument. The light‐curing unit tip was positioned perpendicular to and in direct contact with the top surface of the polyethylene tube (0 mm from the composite surface). All composites were light‐cured for 40 s at an irradiance of 1000 mW/cm^2^ using the same LED light‐curing unit (VALO Grand Cordless) and irradiance employed for adhesive polymerization. The experimental setup is illustrated in Figure 1.

**Table 1:** Composite resins used in this study, with respective composition and filler content.

Brand (manufacturer)	Composition	Filler content
G‐aenial Universal Flo (GC Corp, Tokyo, Japan)	UDMA, TEGDMA, Bis‐MEPP, silicon dioxide, and strontium glass (nanohybrid)	69 wt% 50 vol%
Beautifil Injectable X SL (Shofu, Kyoto, Japan)	Bis‐GMA, TEGDMA, BIS‐EMA, barium aluminosilicate glass, and silicon dioxide nanoparticles	72 wt% 58 vol%
Estelite Flow Quick (Tokuyama, Tokyo, Japan)	Bis‐GMA, Bis‐EMA, TEGDMA, silanized ceramic particles, silica, and zirconium oxide (nanoparticles)	71 wt% 46 vol%
Filtek Supreme XT (3 M ESPE, St. Paul, MN, USA)	Bis‐GMA, Bis‐EMA, UDMA, TEGDMA, zirconia‐silica clusters, and silica nanoparticles	78.5 wt% 63.3 vol%

### 2.1 Microshear Bond Strength Testing

After 48 h of storage in distilled water at 37°C, the polyethylene tubes were carefully removed using a scalpel blade to expose the composite cylinders. Each PVC ring was individually attached to a shear‐testing jig mounted in a universal testing machine (EMIC DL2000, Instron Corp., São José dos Pinhais, PR, Brazil). A blade attached to the upper part of the machine was positioned at the base of the composite cylinder. The enamel/composite interface was tested under shear mode until failure at a crosshead speed of 0.5 mm/min. The load to failure (N) was divided by the bonding surface area (mm^2^) of the specimen to determine microshear bond strength (μSBS) in MPa.

### 2.2 Failure Mode Analysis

The specimens were examined under a stereomicroscope at ×40 magnification, and the failure modes were categorized as “adhesive” (between enamel and composite), “cohesive in composite,” “cohesive in enamel,” or “mixed” (both cohesive and adhesive). Pre‐test failures were not removed from the group and were assigned a value of zero.

Representative specimens were dried in a desiccator, sputter‑coated with Au–Pd, and observed under a scanning electron microscope (VEGA 3 LMU, Tescan Group, Brno‐Kohoutovice, Czech Republic) at ×1000 and ×10,000 magnifications.

### 2.3 Statistical Analysis

Since the data showed normal distribution (Shapiro–Wilk test, *p* > 0.05) and homogeneity of variance (Levene's test, *p* > 0.05), the effects of the experimental factors were analyzed using a three‐way ANOVA. Multiple comparisons were performed using Tukey's post‐hoc test. Partial eta squared (*ηp* ^2^) was calculated as a measure of effect size for each factor. Statistical analysis of failure modes was conducted using Fisher's exact test and the Chi‐square test. All analyses were conducted at a significance level of 5% using statistical software (SPSS version 26.0; IBM Corp., Chicago, IL, USA).

## 3 Results

The analysis demonstrated statistically significant main effects for bonding strategy (*p* < 0.001), but not for composite resin (*p* = 0.044), and enamel preparation (*p* = 0.042). A significant interaction was observed for composite resin × bonding strategy (*p* = 0.008). In contrast, no significant interactions were found for interactions between composite resin × enamel preparation (*p* = 0.299), enamel preparation × bonding strategy (*p* = 0.064), and composite resin × enamel preparation × bonding strategy (*p* = 0.078). The effect size of the factors differed markedly, with bonding strategy showing a very large effect size (*ηp* ^2^ = 0.758), whereas composite type (*ηp* ^2^ = 0.054), enamel preparation (*ηp* ^2^ = 0.029), and composite resin × bonding strategy interaction (*ηp* ^2^ = 0.079) showed small effects.

**Figure 2:**
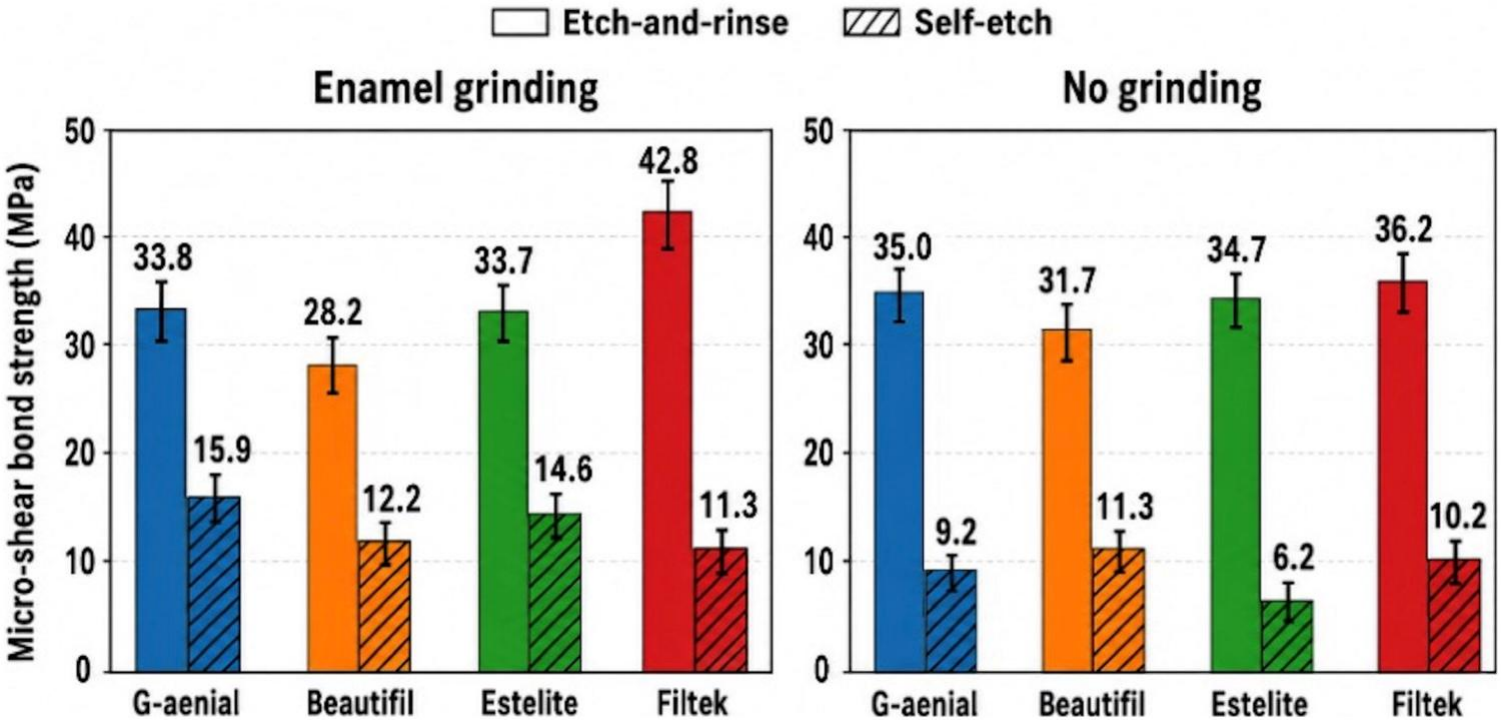
Mean microshear bond strength values (MPa) according to composite resin, enamel preparation, and adhesive strategy. Bars represent means and error bars indicate 95% confidence intervals.

Table 2 and Figure 2 present the mean microshear bond strength for all the experimental groups. The etch‐and‐rinse strategy resulted in significantly higher bond strength than the self‐etch approach, irrespective of the enamel preparation, for all the resin composites (*p* < 0.05). Comparisons among composite resins under each experimental condition revealed significantly lower bond strength for Beautifil Injectable X compared with Filtek Supreme XT when grinding and acid etching were performed (*p* = 0.021).

**Table 2:** Mean microshear bond strength (standard deviation) values according to composite type, enamel grinding, and adhesive strategy.

Composite	Enamel grinding		No grinding	
	Etch‐and‐rinse	Self‐etch	Etch‐and‐rinse	Self‐etch
G‐aenial	33.77 (5.40) ABa	15.90 (7.04) Ab	35.01 (8.49) Aa	9.25 (4.64) Ab
Beautifil	28.19 (8.39) Ba	12.25 (6.41) Ab	31.71 (7.69) Aa	11.35 (3.71) Ab
Estelite	33.70 (5.17) ABa	14.56 (5.66) Ab	34.66 (8.45) Aa	6.20 (5.25) Ab
Filtek	42.79 (6.09) Aa	11.33 (5.51) Ab	36.24 (11.45) Aa	10.18 (6.65) Ab

*Note:* Means followed by different uppercase letters within columns and different lowercase letters within rows differ significantly (Tukey's test, *p* < 0.05).

Failure‐mode distribution is presented in Table 3. Adhesive failures were the most frequent failure mode (35%). Within the self‐etch groups, adhesive failures occurred in 70% of specimens (56/80), whereas no adhesive failures were observed in the etch‐and‐rinse groups (0/80 specimens). Enamel grinding significantly affected the occurrence of adhesive failures within the self‐etch groups, reducing their frequency from 85.0% (34/40) in unground enamel to 55.0% (22/40) in ground enamel (*p* = 0.007). Composite resin type did not significantly influence the frequency of adhesive failures (*p* = 0.883).

**Table 3:** Failure mode distribution (%) in function of enamel grinding, adhesive strategy, and composite.

Enamel grinding	Adhesive strategy	Resin composite	Adhesive failure (%)	Cohesive in composite (%)	Mixed failure (%)	Pre‐test failure (%)
Yes	Etch‐and‐rinse	G‐aenial	0	40	60	0
		Beautifil	0	0	100	0
		Estelite	0	0	100	0
		Filtek	0	10	90	0
		G‐aenial	60	0	40	0
Yes	Self‐etch	Beautifil	60	0	30	10
		Estelite	60	0	40	0
		Filtek	40	0	60	0
		G‐aenial	0	10	90	0
No	Etch‐and‐rinse	Beautifil	0	0	100	0
		Estelite	0	0	100	0
		Filtek	0	20	80	0
		G‐aenial	70	0	20	10
No	Self‐etch	Beautifil	100	0	0	0
		Estelite	70	0	10	20
		Filtek	100	0	0	0

*Note:* Obs: No cohesive failures in enamel were observed.

Four specimens (2.5%) debonded totally or partially during removal of the polyethylene tubes, but were not excluded from the analysis. Their recorded values (0.0–1.3 MPa) were retained and the failure mode was classified as pre‐test.

**Figure 3:**
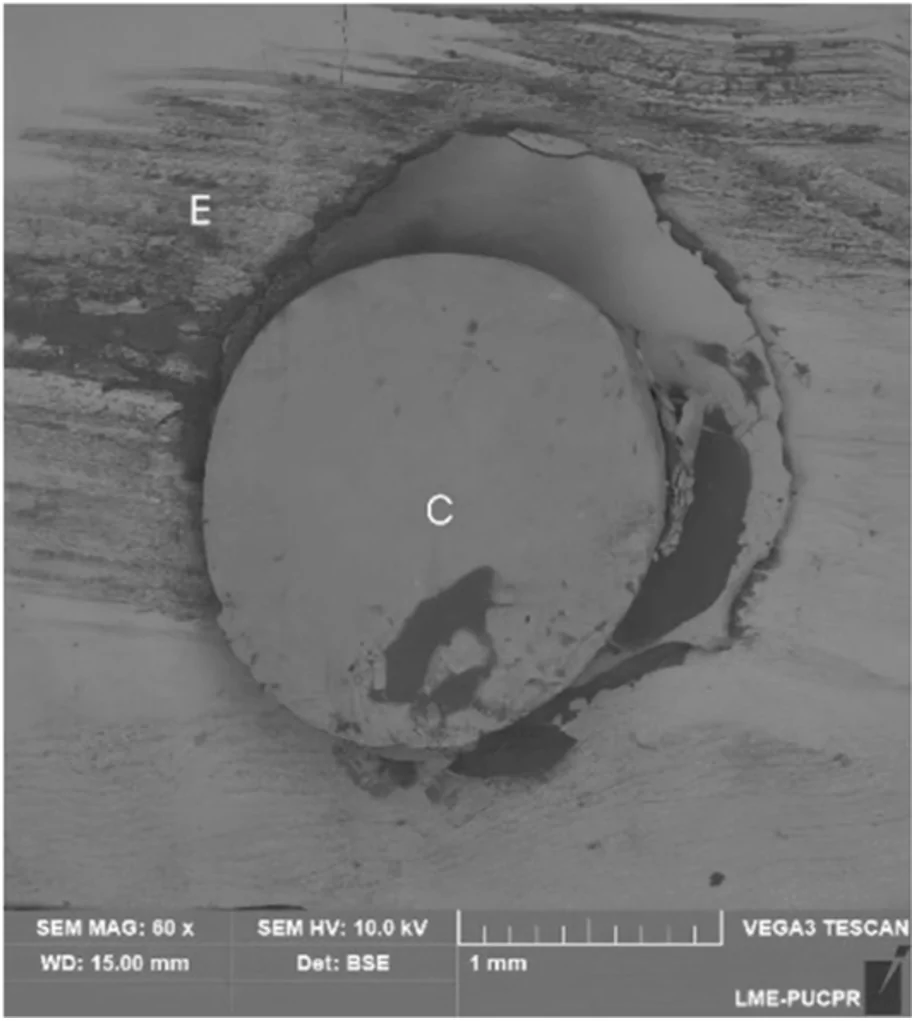
SEM micrograph (BSE mode, ×60) showing cohesive failure within the composite (C) with intact enamel (E).

**Figure 4:**
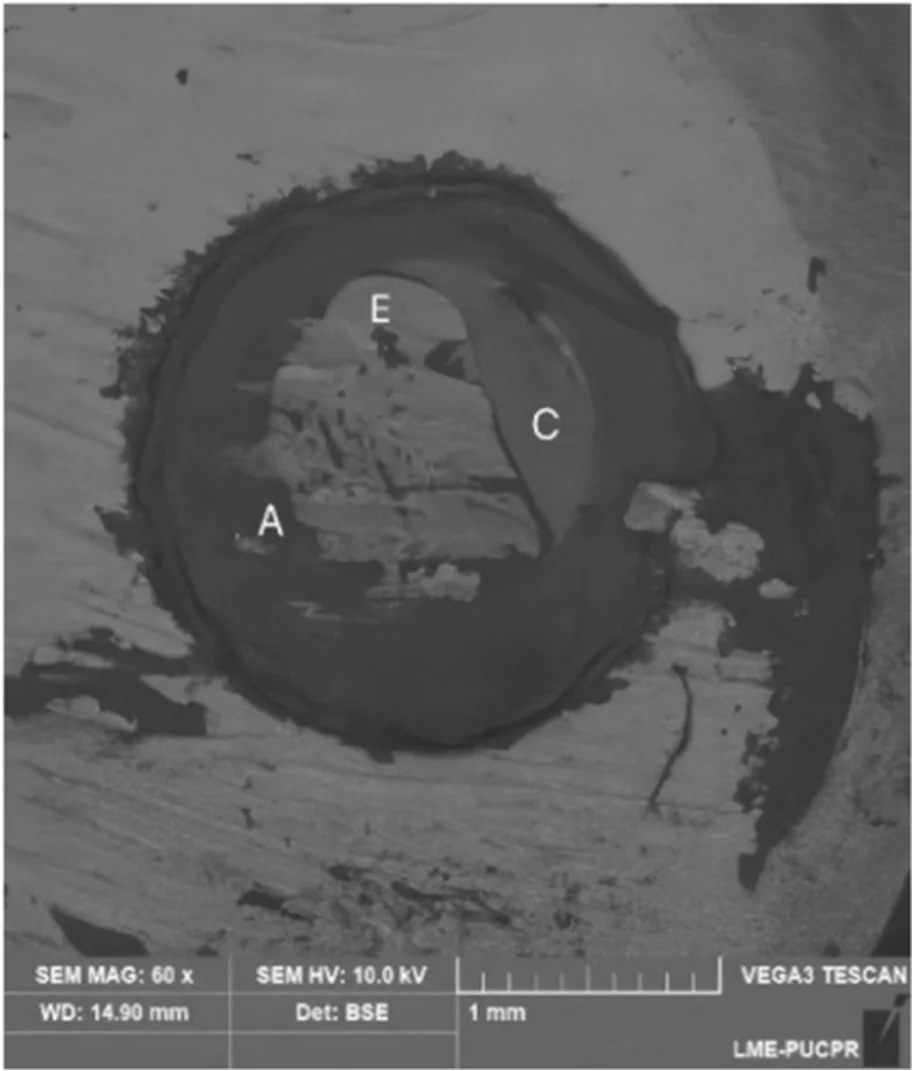
SEM micrograph (BSE mode, ×60) showing mixed failure involving enamel (E), adhesive (A), and composite (C).

**Figure 5:**
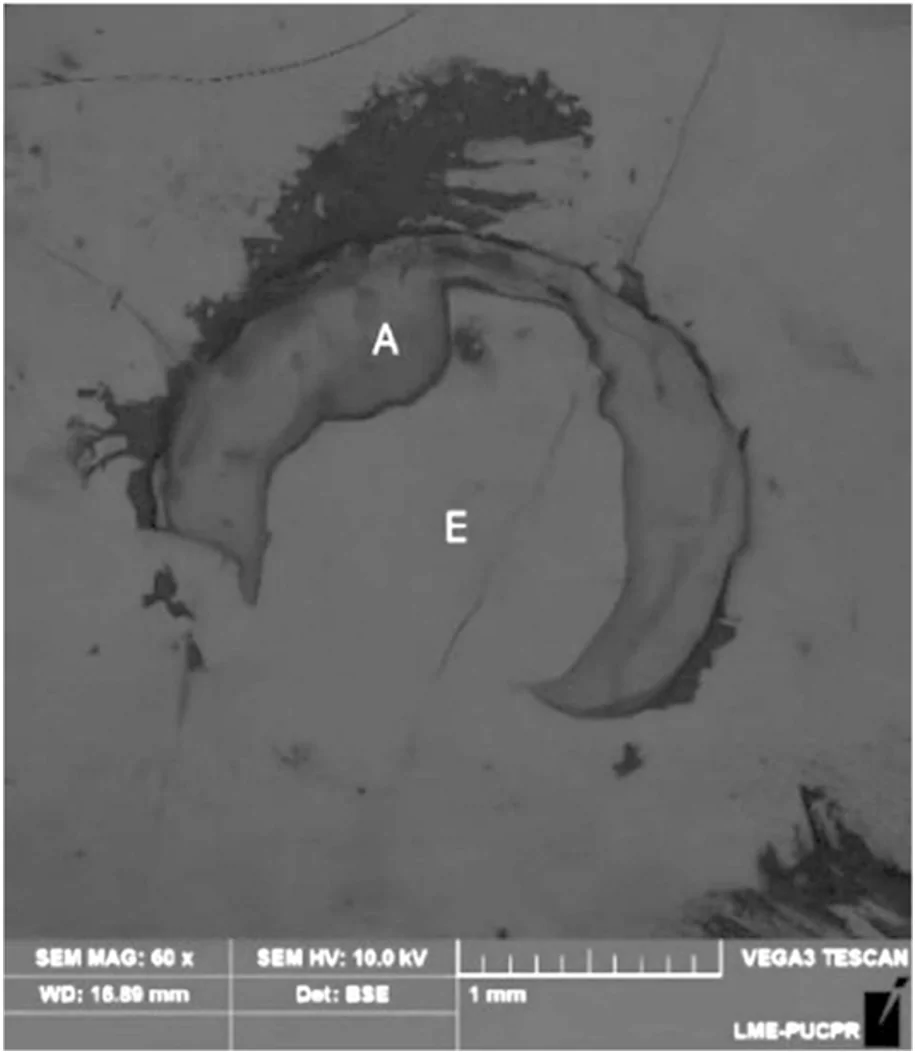
SEM micrograph (BSE mode, ×60) demonstrating adhesive failure at the enamel–adhesive interface (E/A).

**Figure 6:**
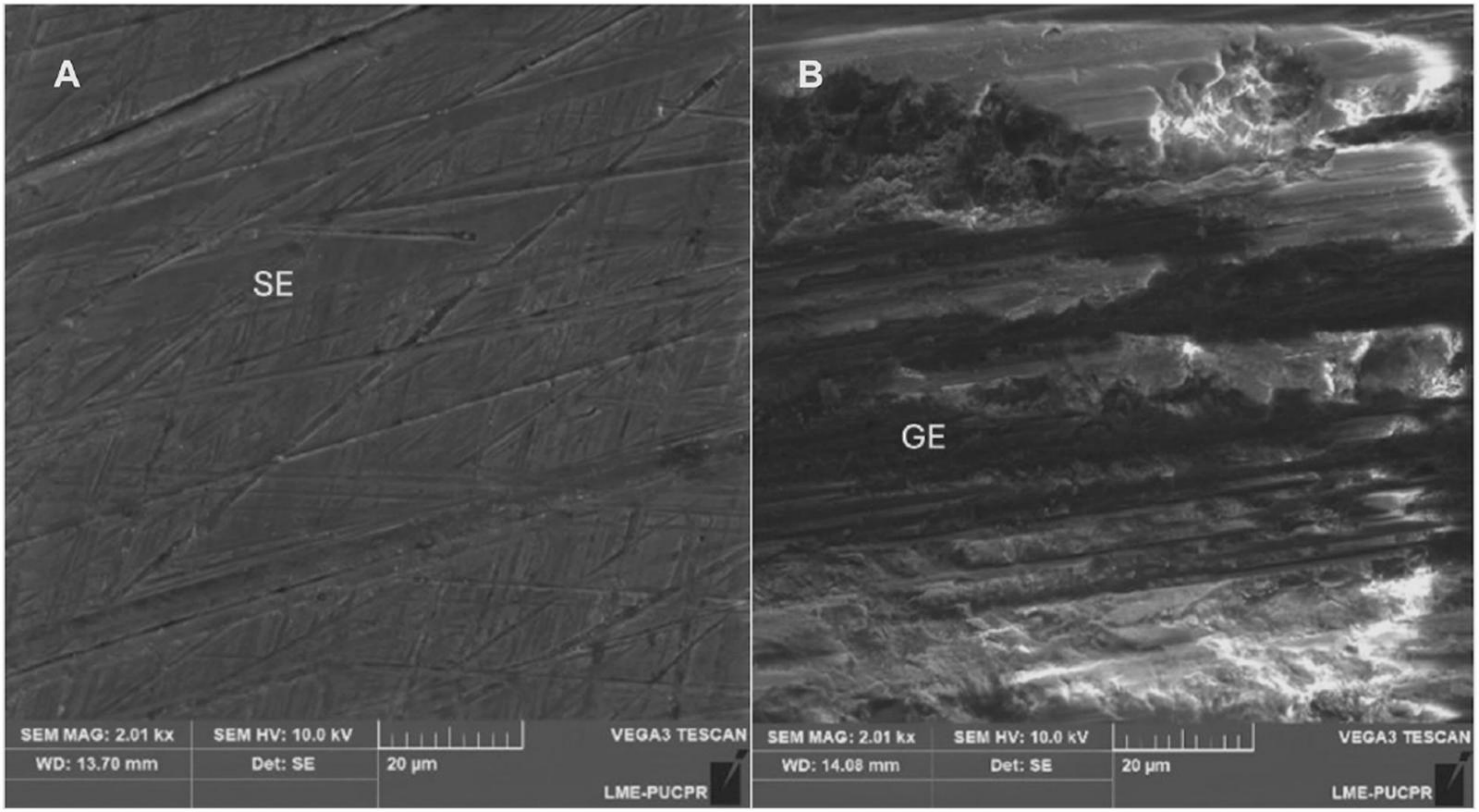
SEM micrographs (×2000) of enamel surfaces: (A) sound enamel (SE) without grinding; (B) ground enamel (GE) after diamond bur preparation.

Figures 3, 4, 5 show representative SEM images of the different failure modes. Figure 6a illustrates the surface morphology of sound enamel, whereas Figure 6b depicts enamel surfaces subjected to diamond bur grinding, demonstrating marked morphological differences between both conditions.

## 4 Discussion

This study evaluated the influence of enamel surface preparation and adhesive strategy on the microshear bond strength of highly filled injectable composite resins compared with a conventional nanofilled composite. The results demonstrated that all three factors significantly affected enamel bond strength, and the three null hypotheses were therefore rejected. However, the magnitude of these effects differed markedly: bonding strategy showed a very large effect size (*ηp* ^2^ = 0.758), whereas composite type (*ηp* ^2^ = 0.054) and enamel preparation (*ηp* ^2^ = 0.029) showed small effects. Therefore, although statistically significant, the influence of composite type and enamel preparation on bond strength was of limited magnitude compared with that of the bonding strategy.

A significant interaction between composite resin and adhesive strategy was also observed, indicating that the bonding performance of the tested materials depended on the adhesive approach employed. Although all composites demonstrated higher bond strength when the etch‐and‐rinse strategy was applied, the magnitude of this improvement varied among materials. These differences may be related to variations in filler content, resin matrix composition, and viscosity, which can influence the interaction between the composite material and the adhesive interface, particularly when self‐etch approaches are used.

The viscosity of injectable composites represents an important factor that may affect their clinical performance. Limited flowability over the substrate surface can impair wetting (de Andrade et al. 2007), which may partially explain the lower bond strength observed for Beautifil Injectable compared with Filtek Supreme XT in the ground/etch‐and‐rinse condition, the only combination in which a significant difference between composites was detected. Among the injectable composites tested, Beautifil Injectable X SL presented the highest volumetric filler content (58 vol%, against 50 vol% for G‐aenial Universal Flo and 46 vol% for Estelite Flow Quick; Table 1), and a previous investigation also reported higher viscosity values for Beautifil Flow Plus, a material with similar characteristics to Beautifil Injectable, compared with other flowable composites (Al‐Saud 2021). Filler content alone, however, does not account for the pattern observed, since G‐aenial Universal Flo and Estelite Flow Quick differed in volumetric filler content yet did not differ significantly in any experimental condition. Increased viscosity may not only hinder injection but may also increase the risk of incomplete adaptation and void formation (Alqudaihi et al. 2019), which can negatively affect the mechanical behavior of restorative materials (Ferracane and Lawson 2021).

Nevertheless, these findings should be interpreted with caution and cannot be directly extrapolated to clinical conditions. In the present experimental model, all composites were built up in polyethylene tubes with a 1‐mm bonding diameter. The injectable composites were delivered directly through their metallic needle tips, whereas the conventional composite was placed and condensed with a fine instrument. Material viscosity and insertion technique were therefore confounded, and the present design does not allow their individual contributions to the bonding outcome to be determined.

Consistent with previous studies evaluating universal adhesives (Sai et al. 2018; Erickson et al. 2009), total enamel etching with phosphoric acid resulted in significantly higher bond strength than the self‐etch strategy. This superiority is attributed to the more pronounced etching pattern produced by phosphoric acid, which removes the aprismatic enamel layer and creates microporosities that promote micromechanical interlocking (Takeda et al. 2019; Sano et al. 1999). In contrast, self‐etch adhesives typically produce shallower demineralization patterns with limited interprismatic dissolution, which may restrict resin infiltration and compromise bonding effectiveness (Van Meerbeek et al. 2010).

These findings are particularly relevant for restorative procedures performed using injectable composite techniques. In such approaches, restorations are often placed on intact enamel surfaces without prior mechanical preparation. Under these conditions, the absence of phosphoric acid etching may compromise adhesion, highlighting the importance of this approach when universal adhesives are used in combination with highly filled injectable composites.

Although a statistically significant main effect of enamel preparation was detected, pairwise comparisons revealed no significant differences between ground and unground enamel within any bonding strategy, indicating an effect of limited magnitude. The morphological differences between sound and prepared enamel have been widely described in the literature (Kanemura et al. 1999; Mine et al. 2010; Pashley and Tay 2001; Pivetta et al. 2008). Sound enamel contains an acid‐resistant aprismatic layer that may reduce susceptibility to demineralization (Bourgi et al. 2024; Mine et al. 2010). Consequently, less homogeneous etching patterns may occur, particularly when mild acids are used, such as those incorporated in some universal adhesives (Pashley and Tay 2001; Pivetta et al. 2008).

The presence of a smear layer in prepared substrates (Bortolotto et al. 2009) and the differences in acid‐etch resistance between aprismatic and prismatic enamel require careful optimization of enamel surface conditions for effective bonding (Pivetta et al. 2008). Diamond bur roughening has been proposed as a complementary strategy, particularly for mild self‐etch adhesive systems (Hoshika et al. 2018; Mine et al. 2010; Van Meerbeek et al. 2020). In the present study, the universal adhesive used presents an ultra‐mild acidity (pH 2.7), which may have limited its demineralization capacity on enamel (Cuevas‐Suárez et al. 2019; Mine et al. 2010). Although surface roughening increases the available bonding area and removes the aprismatic enamel layer, these effects were insufficient to compensate for the limited etching potential of the universal adhesive when applied in self‐etch mode.

Etch‐and‐rinse has been consistently reported as a more effective bonding approach when universal adhesives are employed (Cuevas‐Suárez et al. 2019). Phosphoric acid removes the smear layer, increases surface energy, and produces a more homogeneous etching pattern (Tiznado‐Orozco et al. 2015). Furthermore, it enhances micromechanical interlocking and may facilitate chemical interactions between acidic functional monomers and hydroxyapatite, thereby improving enamel bonding (Tsujimoto et al. 2016).

In the present study, enamel grinding significantly reduced the occurrence of adhesive failures in self‐etch mode, from 85% in unground enamel to 55% in ground enamel. No adhesive failures occurred in any etch‐and‐rinse group, regardless of enamel grinding. This dissociation between failure mode and bond strength, with grinding altering the former without producing pairwise differences in the latter, suggests that removing the aprismatic layer improved the quality of the adhesive interface even when the resulting increase in µSBS was too small to reach statistical significance. A comparable dissociation has been reported for mild self‐etch systems applied to bur‐cut enamel (Hoshika et al. 2018; Mine et al. 2010).

Four specimens, all belonging to self‐etch groups, were classified as pre‐test failures. Such failures are commonly reported in microshear bond strength testing and reflect difficulties inherent to the method, including the small bonded area, the manipulation required to remove the polyethylene tubes, and the fragility of interfaces produced under unfavorable bonding conditions (Van Meerbeek et al. 2010). These specimens were retained in the analysis, since their exclusion would have artificially increased the mean values of those groups with the poorest bonding performance.

The sample size calculation was based on an assumed standard deviation of 2.0 MPa, whereas the standard deviations observed in the present study ranged from 3.7 to 11.5 MPa. This limited sensitivity is consistent with the small effect sizes obtained for composite type and enamel preparation, and with the absence of pairwise differences between ground and unground enamel. Larger samples would be required to characterize these two factors more precisely.

Despite these limitations, the present findings indicate that universal adhesives should preferably be used in the etch‐and‐rinse approach when bonding to enamel, particularly in restorative procedures involving large enamel surfaces. Although enamel grinding is not routinely performed in mock‐up or injection techniques, it reduced the occurrence of adhesive failures in self‐etch mode. Whether this translates into improved clinical retention remains to be determined.

## 5 Conclusions

Within the limitations of this study, the etch‐and‐rinse strategy improved enamel bond strength and eliminated adhesive failures for all composites tested, regardless of enamel grinding. When the universal adhesive was applied in self‐etch mode, prior enamel grinding with a diamond bur reduced, but did not eliminate, the occurrence of adhesive failures.

## Author Contributions

**João Daniel Paganella Chaves:** investigation, acquisition, interpretation of data, drafting, writing. **Elisa Souza Camargo:** manuscript final review. **Rodrigo Nunes Rached:** analysis of data, methodology. **Evelise Machado de Souza:** conception and design, supervision, writing, review.

## Funding

The authors have nothing to report.

## Ethics Statement

The study protocol was approved by the Research Ethics Committee of the Pontifícia Universidade Católica do Paraná under the code number CAAE: 68127723.2.0000.0020. The document was attached as an Ethical Form.

## Conflicts of Interest

The authors declare no conflicts of interest.

## Data Availability

The data that support the findings of this study are available at 10.5281/zenodo.22167748.
